# Utility of N-Bromosuccinimide for the Titrimetric and Spectrophotometric Determination of Famotidine in Pharmaceutical Formulations

**DOI:** 10.1155/2011/581372

**Published:** 2011-06-12

**Authors:** O. Zenita, K. Basavaiah

**Affiliations:** Department of Chemistry, University of Mysore, Manasagangothri, Mysore 570 006, India

## Abstract

Two titrimetric and two spectrophotometric methods are described for the assay of famotidine (FMT) in tablets using N-bromosuccinimide (NBS). The first titrimetric method is direct in which FMT is titrated directly with NBS in HCl medium using methyl orange as indicator (method A). The remaining three methods are indirect in which the unreacted NBS is determined after the complete reaction between FMT and NBS by iodometric back titration (method B) or by reacting with a fixed amount of either indigo carmine (method C) or neutral red (method D). The method A and method B are applicable over the range of 2–9 mg and 1–7 mg, respectively. In spectrophotometric methods, Beer's law is obeyed over the concentration ranges of 0.75–6.0 *μ*g mL^−1^ (method C) and 0.3–3.0 *μ*g mL^−1^ (method D). The applicability of the developed methods was demonstrated by the determination of FMT in pure drug as well as in tablets.

## 1. Introduction

**Figure 1:**
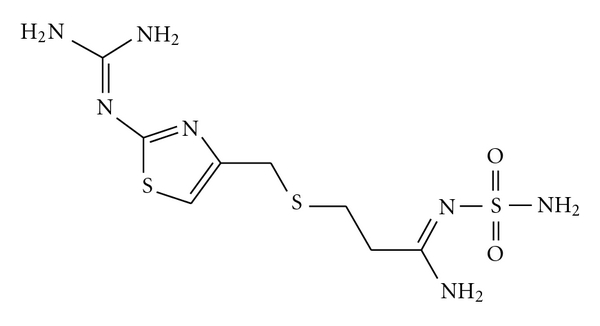
Structure of famotidine.

Famotidine (FMT), 3-[2-(diaminomethyleneamino)thiazol-4-ylmethylthio]-N-sulfamoylpropionamidine (Figure 1), is a histamine H_2_-receptor antagonist (H_2_-RA). It is widely used for the treatment of duodenal ulcers, benign gastric ulcer, reflux oesophagitis, and hyperacid secretory conditions. FMT is official in both the British Pharmacopoeia (BP) [1] and the United States Pharmacopoeia (USP) [2]. The BP [1] recommends thin-layer chromatography using a silica gel F_254_ precoated plate (Fischer Silica Gel GF plates are suitable) and a mixture of 2 volumes of 13.5 M ammonia, 20 volumes of toluene, 25 volumes of methanol, and 40 volumes of ethyl acetate as the mobile phase. The USP [2] recommends a potentiometric nonaqueous method for the determination of FMT using perchloric acid as the titrant and a HPLC method using a mixture of acetate buffer of pH 6 : acetonitrile (93 : 7) as a mobile phase with UV detection at 275 nm. 

Several procedures have been reported in the literature for the analysis of FMT. The reported methods include HPLC [3–5], HPTLC [6, 7], capillary electrophoresis [8], potentiometry [9], differential pulse voltammetry [10], spectrofluorimetry [11, 12], polarography [13], and UV-spectrophotometry [14]. Some of these methods involve several manipulation steps, which are not simple for routine analysis of pharmaceutical formulations and need sophisticated instruments.

Titrimetry and visible spectrophotometry may serve as useful alternatives to many of the aforesaid sophisticated techniques because of their cost effectiveness, ease of operation, sensitivity, remarkable accuracy and precision, and wide applicability. From the literature survey, it is revealed that two titrimetric methods have been reported using chloramine-T [15] and potassium iodate [16] as oxidimetric reagents. Visible spectrophotometric methods based on diverse reaction chemistry have been proposed for the assay of FMT in pharmaceuticals. Extractive spectrophotometric procedures [17] based on ion pair complexation reaction with bromocresol green (BCG) and bromothymol blue (BTB) have been used for the assay of FMT. Based on the formation of charge-transfer complex, some *π* acceptors such as chloranil, dichlorodicyanobenzoquinone and dichloronitrophenol [18], tetracyanoquinodimethane [19], and *p*-chloranilic acid [20, 21] have been employed for its determination in commercial tablet formulations. Other visible spectrophotometric methods based on different reactions such as complex formation reaction with copper (II) chloride in methanolic medium [22], cupric acetate [23], palladium (II) chloride in Britton Robinson buffer solution in the pH range 2.23–8.5 [24], condensation of amino group in FMT with ninhydrin in DMF medium [25], formation of orange-colored product with sodium nitroprusside in alkaline medium [26], bromination of FMT with brominating mixture [27] reduction of Folin-Ciocalteau reagent [28], and oxidation of FMT by Fe (III) [29] or NBS [30] were reported for the assay of FMT. Most of the above visible spectrophotometric methods suffer from one or another disadvantage such as the use of organic solvents [17–22, 25], poor sensitivity [18, 20–23, 26, 27], less selective [28], use of expensive reagents [19], use of heating step [25, 29], narrow linear range [25, 30], and close pH control [17, 24], as indicated in Table 1. The present investigation aims to develop simple, sensitive, and cost-effective methods for the determination of FMT in pure form and in dosage forms using titrimetric and spectrophotometric techniques. The methods utilized NBS, indigo carmine, and neutral red as reagents. The developed methods offer the advantages of simplicity, speed, accuracy, and precision without the need for costly equipment/chemicals.

## 2. Experimental

### 2.1. Apparatus

All absorbance measurements were made on a Systronics model 106 digital spectrophotometer (Ahmedabad, India) provided with 1 cm matched quartz cells.

### 2.2. Materials and Reagents

All chemicals and reagents used were of analytical or pharmaceutical grade.

#### 2.2.1. N-Bromosuccinimide (NBS)

0.05 M NBS solution was prepared by dissolving 8.9 g of chemical (Loba Chemie, Mumbai, India) in water with the aid of heat and diluted to 1 litre with water and standardized [31]. The solution was stored in amber-colored bottle and diluted appropriately to 0.01 M and 0.02 M NBS for method A and method B, respectively. The NBS solution was further diluted to get 150 *μ*g mL^−1^  and 200 *μ*g mL^−1^ for use in method C and method D, respectively.

#### 2.2.2. Sodium Thiosulphate (0.04 M)

Prepared by dissolving 9.93 g of chemical (S. D. Fine Chem. Ltd., Mumbai, India) in one liter of water for use in titrimetric method B.

#### 2.2.3. Sulphuric Acid and HCl

Concentrated sulphuric acid (S. D. Fine Chem, Mumbai, India, sp. gr. 1.84) and concentrated HCl (S. D. Fine Chem, Mumbai, India, sp. gr. 1.18) was appropriately diluted with water to get the required concentrations.

#### 2.2.4. Potassium Iodide

A 10% solution was prepared by dissolving 10 g of the chemical in 100 mL of water and used in titrimetric method B.

#### 2.2.5. Starch Indicator

One g of the reagent (Merck, Mumbai, India) was made into a paste and poured into 100 mL of boiling water, boiled for 1 min and cooled, and used for titrimetric method B.

#### 2.2.6. Indigo Carmine (200 *μ*g mL^−1^) and Neutral Red (240 *μ*g mL^−1^)

The dye solutions were prepared by dissolving calculated quantity of the chemicals, indigo carmine (S. D. Fine Chem, Mumbai, India, dye content 90%) and neutral red (S. D. Fine Chem, Mumbai, India, dye content 90%) in water and filtered.

#### 2.2.7. Standard FMT Solution

Pharmaceutical grade FMT certified to be 99.98% pure was received as a gift from Cipla India Ltd, Mumbai, India, and used as received. Standard FMT solution (1 mg mL^−1^, 15 *μ*g mL^−1^, and 6 *μ*g mL^−1^) was prepared by dissolving calculated quantity of pure drug in 0.1 M HCl (method A, method C, and method D) and in 0.5 M H_2_SO_4_ (method B).

#### 2.2.8. Pharmaceutical Preparations

Two brands of tablets containing FMT, Topcid-20 (Torrent Pharmaceuticals Ltd., H. P, India), and Famocid-20 (Sun Pharmaceuticals Industries, Jammu, India), used in the investigation, were purchased from local commercial sources.

### 2.3. Materials and Reagents Used in Reference Method

#### 2.3.1. Apparatus

An Elico 120 digital pH meter provided with a combined glass-SCE electrode system (Equip-Tronics, Mumbai, India) was used for potentiometric titration.

#### 2.3.2. Preparation of Modified Glass-Saturated Calomel Electrode

Aqueous potassium chloride solution contained in the saturated calomel electrode was completely removed, and the electrode was rendered anhydrous and filled with 0.1 N lithium perchlorate in acetic anhydride.

#### 2.3.3. Perchloric Acid (0.1 M)

The commercially available 0.1 M perchloric acid (Merck, Mumbai, India) was standardized against 0.1 M potassium dihydrogen phthalate [32].

**Lithium Perchlorate (0.1 N)**

The solution was prepared by dissolving calculated quantity of lithium perchlorate (Himedia Lab. Pvt. Limited, Mumbai, India) in acetic anhydride (Merck, Mumbai, India).

### 2.4. General Procedure

#### 2.4.1. Titrimetry

**Method A**

Different volumes (2–9 mL) of standard solution containing 1 mg mL^−1^ FMT were accurately measured and transferred into a 100 mL titration flask, and the volume was made up to 10 mL with 0.1 M HCl. Five mL of 2 M HCl and one spatula of KBr were added. The resulting solution was titrated against 0.01 M NBS using 2 drops of methyl orange as indicator. The titration was carried by dropwise addition of NBS with continuous shaking and  the end point is the disappearance of pink color.

**Method B**

Different volumes (1–7 mL) of standard solution containing 1 mg mL^−1^ FMT were taken in a 100 mL titration flask, and the volume was made up to 10 mL with 0.5 M H_2_SO_4_. Two mL of 5 M H_2_SO_4_ and one spatula of KBr were added into the flask. Ten mL of NBS (0.02 M) was pipetted into the flask; the content was mixed and kept aside for 5 min, then 5 mL of 10% potassium iodide solution was added, and the liberated iodine was titrated against sodium thiosulphate (0.04 M) using starch indicator. A blank titration was performed under identical conditions.

#### 2.4.2. Spectrophotometry

**Method C**

Different aliquots of 0.25, 0.5, 1.0, 2.0, 3.0, and 4.0 mL of standard FMT solution (15 *μ*g mL^−1^) were transferred into a series of 10 mL standard volumetric flasks, and the total volume in each flask was adjusted to 4 mL with 0.1 M HCl. To each flask, 1 mL of 1 M HCl followed by 1 mL of 150 *μ*g mL^−1^ NBS was added. The contents of each flask were mixed well and kept aside for 15 minutes with occasional shaking. One mL of 200 *μ*g mL^−1^ indigo carmine was added, and the volume was made up to the mark with distilled water after 5 min, and the absorbance was measured at 610 nm versus reagent blank prepared in a similar manner.

**Method D**

Different aliquots (0.5, 1.0, 2.0, 3.0, 4.0, and 5.0) mL of standard 6 *μ*g mL^−1^ FMT solution were accurately measured and transferred into a series of 10 mL calibrated flasks by means of a microburette, and the total volume was adjusted to 5 mL with 0.1 M HCl. To each flask, 1 mL of 200 *μ*g mL^−1^ NBS was added using microburette. And the contents of each flask were mixed well and kept aside for 15 minutes. One mL of 240 *μ*g mL^−1^ neutral red was added to each flask and mixed well. The volume was made up to the mark with distilled water after 5 min, and the absorbance was measured at 530 nm versus reagent blank prepared in a similar manner.

Calibration graphs were prepared by plotting the increasing absorbance values versus concentrations of FMT. The concentration of the unknown was read from the respective calibration graph or deduced from the regression equation derived using the Beer's law data.

#### 2.4.3. Procedure for the Assay of FMT in Tablets

Twenty tablets were weighed accurately and ground into a fine powder. A quantity of the powder containing 100 mg of FMT was accurately weighed into a 100 mL calibrated flask, and 60 mL of 0.1 M HCl (titrimetric method A) or 0.5 M H_2_SO_4_ (titrimetric method B) was added. The content was shaken for about 20 min; the volume was diluted to the mark with the respective solvent mixed, and filtered using a Whatman no. 42 filter paper. First, 10 mL portion of the filtrate was discarded, and a convenient aliquot was taken, and the assay was completed according to the titrimetric procedures described earlier. The tablet extract containing FMT at a concentration of 1 mg mL^−1^ was then diluted stepwise with 0.1 M HCl to obtain working concentrations of 15 *μ*g mL^−1^ and 6 *μ*g mL^−1^ in FMT for spectrophotometric method C and method D, respectively. A convenient aliquot was then subjected to analysis by spectrophotometric procedures described above.

#### 2.4.4. Placebo Blank Analysis

A placebo blank of the composition: talc (10 mg), starch (5 mg), acacia (5 mg), methyl cellulose (10 mg), sodium citrate (5 mg), magnesium stearate (10 mg), and sodium alginate (5 mg) was made, and its solution was prepared as described under “Procedure for the assay of famotidine in pharmaceutical preparations” and then subjected to analysis using the procedures described above.

#### 2.4.5. Procedure for the Determination of FMT in Synthetic Mixture

To the placebo blank of the composition described above, 100 mg of FMT was added homogenized, and transferred to a 100 mL calibration flask, and solution was prepared as described under tablets. The solution was mixed well and filtered using a Whatman no. 42 filter paper. The resulting solution was assayed (*n* = 5) by titrimetry according to the procedures described above. The synthetic mixture solution (1 mg mL^−1^ in FMT) was then diluted stepwise with 0.1 M HCl to obtain working concentrations of 15 *μ*g mL^−1^ and 6 *μ*g mL^−1^ in FMT for spectrophotometric method C and method D, respectively. A convenient aliquot was then subjected to analysis by using the procedures described above.

#### 2.4.6. Procedure for Reference Method

Two hundred and fifty mg of FMT was accurately weighed and dissolved in 80 mL of glacial acetic acid and titrated with 0.1 M perchloric acid, using modified glass-saturated calomel electrode which was described above. Near the equivalence point, the titrant was added in 0.05 mL increments, and after each addition of titrant, the solution was stirred for 30 s, and the steady potential was recorded. The titration was continued until there was no significant change in the potential on further addition of the titrant. A blank determination was performed to make necessary correction.

## 3. Results and Discussion

NBS is a brominating and oxidizing agent that is perhaps the most important positive bromine containing organic compound, and it is used for the specific purpose of brominating alkenes at the allylic position [33]. NBS has earlier been used for the assay of FMT using *p*-aminophenol as reagent, and the present work extended the utility of NBS as an oxidimetric as well as brominating agent. Two titrimetric and two spectrophotometric methods to the determination of FMT were developed and validated as per the current ICH guidelines [34].

**Figure 2:**
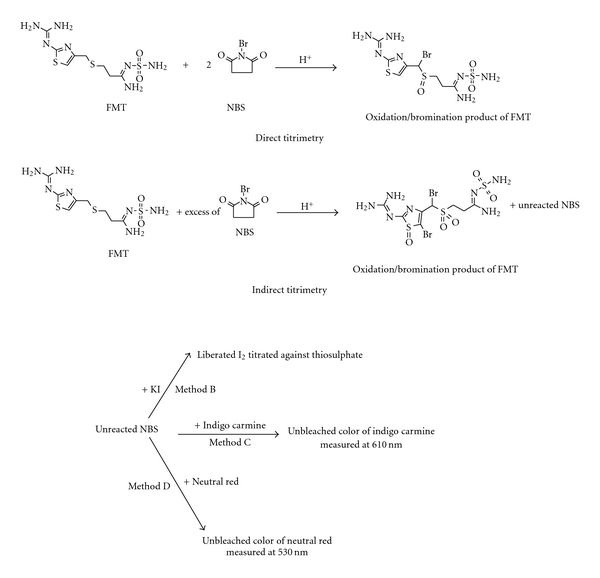
Tentative reaction schemes.

**Figure 3:**
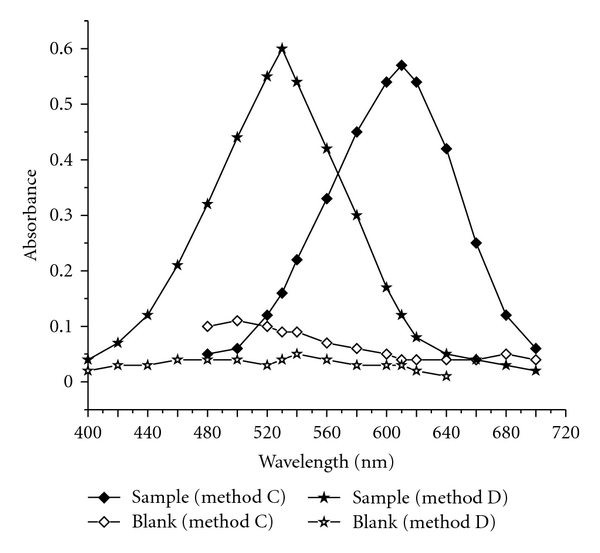
Absorption spectra (4.5 *μ*g mL^−1^ FMT in method C and 1.8 *μ*g mL^−1^ in method D).

The titrimetric method A is direct where the FMT is directly titrated against NBS in HCl medium using methyl orange as indicator, whereas in back titration (method B), the reactants were allowed to stand for some time in H_2_SO_4_ medium, and the unreacted NBS is determined iodometrically (Figure 2). The proposed spectrophotometric methods involve two steps: oxidation/bromination of FMT by NBS (first step) and estimation of unconsumed NBS with indigo carmine (method C) which give maximum absorption at 610 nm (Figure 3) or neutral red (method D) which give maximum absorption at 530 nm (Figure 3) (second step). The tentative reaction scheme is shown in Figure 2.

### 3.1. Optimization of Reaction Conditions

#### 3.1.1. Titrimetry

The reaction stoichiometry was found to be 1 : 2 (FMT : NBS) in method A and 1 : 5 (FMT : NBS) in method B. In method A, titration was carried out instantaneously, and in method B, the reaction between FMT and NBS was kept for 5 min. The difference in the reaction stoichiometry in method A and B is perhaps due to the difference in reaction time and possibility of getting two different reaction products. The structure of FMT features aliphatic sulphur which can easily undergo oxidation to form sulphoxide and sulphone [35, 36] and also bromination at allylic position [33]. On addition to these reactions, the bromination at C5 position of thiazole ring in FMT and oxidation of sulphur in thiazole to sulphoxide [37] are also possible routes. Electrophilic aromatic substitution at C5 of thiazole requires activating groups [38]. The tertiary-amino group present in FMT is a strong activating group, so the bromination of thiazole in FMT is possible. Based on these observations, two possible reaction products are proposed and shown in Figure 2. In both the methods, constant reaction stoichiometry was obtained only in the presence of KBr. One spatula of KBr was found to be optimum to accelerate the oxidation/bromination process. In direct titration, quantitative results were obtained in HCl medium, and the reaction stoichiometry was unaffected in the concentration range of 0.53–0.8 M HCl. Hence, 5 mL of 2 M HCl in a total volume of 15 mL was fixed. In indirect titration, 2 mL volume of 5 M H_2_SO_4_  in a total volume of 26 mL was found adequate although 1–3 mL resulted in the same value of “*n*.” The oxidation/bromination reaction was found to be complete and quantitative in 5 min, and contact time upto 20 min had no effect on the stoichiometry or the results. Hence, it is necessary to terminate the oxidation/bromination step at the end of 5 min to obtain accurate and precise results. A 10 mL aliquot of 0.02 M NBS solution was found adequate for quantitative oxidation/bromination of FMT in the range determined to be 1–7 mg.

#### 3.1.2. Spectrophotometry

Preliminary experiments were performed to determine the maximum concentration of indigo carmine or neutral red in the acid medium employed, and this was found to be 20 *μ*g mL^−1^ and 24 *μ*g mL^−1^ for indigo carmine and neutral red, respectively. NBS concentration of 15 *μ*g mL^−1^ was found optimum to bleach the blue color due to 20 *μ*g mL^−1^ indigo carmine, whereas in the case of neutral red, 20 *μ*g mL^−1^ NBS was sufficient to destroy the pink color of 24 *μ*g mL^−1^ neutral red. Hence, different amounts of FMT reacted with 15 *μ*g mL^−1^ NBS in method C and 20 *μ*g mL^−1^ NBS in method D. 

 Hydrochloric acid was the ideal medium for the oxidation/bromination of FMT by NBS as well as the latter's determination employing either dye. In method C, the reaction between FMT and NBS was unaffected when 0.5–2.0 mL of 1 M HCl in a total volume of 10 mL was used. Hence, 1 mL of 1 M HCl was used for both steps in method C, whereas in method D, the reaction was found to be faster in lower acid concentration. Hence, 0.1 M HCl which was used to dilute the series of drug solution upto 5 mL was found sufficient for both steps. For a quantitative reaction between FMT and NBS, a contact time of 15 min was found necessary in both the methods C and D; and constant absorbance readings were obtained when contact times were extended upto 60 min. In both the methods, a standing time of 5 min was necessary for the bleaching of the dye color by the residual NBS, and the measured color was found to be stable for several hours in the presence of the reaction product/s.

#### 3.1.3. Methodology of the Reference Method

The method is based on the neutralization reaction of the weak base FMT with perchloric acid as a titrant in glacial acetic acid medium. When a strong acid, such as perchloric acid, is dissolved in a weaker acid, such as acetic acid, the acetic acid is forced to act as a base and accept a proton from the perchloric acid forming an onium ion [39]. The formed onium ion (CH_3_COOH_2_^+^) can very readily give up its proton to react with FMT, so basic properties of the drug are enhanced, and hence titration between FMT and perchloric acid was accurately carried out using acetic acid as solvent.

### 3.2. Method Validation Procedures

The proposed methods have been validated for linearity, sensitivity, precision, accuracy, selectivity, and recovery.

#### 3.2.1. Linearity and Sensitivity

**Figure 4:**
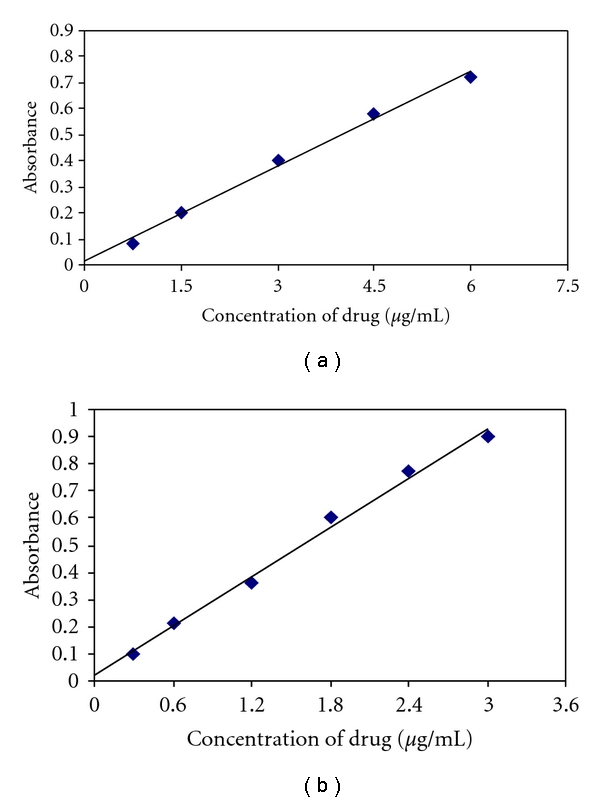
Calibration curves (method C and method D).

Over the range investigated (2–9 mg in method A and 1–7 mg in method B), fixed stoichiometry of 1 : 2 (FMT : NBS) and 1 : 5 (FMT : NBS) in method A and method B, respectively, was obtained in titrimetry which served as the basis for calculations. In spectrophotometry, under optimum conditions, a linear relation was obtained between absorbance and concentration of FMT in the range of  0.75–6.0 ***μ***g mL^**−1**^ (method C) and 0.3–3.0 ***μ***g mL^**−1**^ (method D) (Figure 4). The calibration graph is described by the equation

(1) Y=a+bX,

(where *Y* = absorbance, *a* = intercept, *b* = slope, and *X* = concentration in *μ*g mL^−1^) obtained by the method of least squares. Correlation coefficient, intercept, and slope for the calibration data are summarized in Table 2. Sensitivity parameters such as apparent molar absorptivity and Sandell sensitivity values and the limits of detection and quantification are calculated as per the current ICH guidelines [34] which are compiled in Table 2 that speaks of the excellent sensitivity of the proposed method. The limits of detection (LOD) and quantification (LOQ) were calculated according to the same guidelines using the formulae

(2) $$\text{LOD}=3.3\frac{\sigma }{s}\text{,}\;\;\text{LOQ}=10\frac{\sigma }{s},$$

where *σ* is the standard deviation of five reagent blank determinations, and *s* is the slope of the calibration curve.

#### 3.2.2. Precision and Accuracy

Intraday precision and accuracy of the proposed methods were evaluated by replicate analysis (*n* = 5) of calibration standards at three different concentration levels in the same day. Interday precision and accuracy were determined by assaying the calibration standards at the same concentration levels on five consecutive days. Precision and accuracy were based on the calculated relative standard deviation (RSD, %) and relative error (RE, %) of the found concentration compared to the theoretical one, respectively (Table 3).

#### 3.2.3. Selectivity

The proposed methods were tested for selectivity by placebo blank and synthetic mixture analyses. A convenient aliquot of the placebo blank solution prepared as described earlier was subjected to analysis by titrimetry and spectrophotometry according to the recommended procedures. In all the cases, there was no interference by the inactive ingredients.

 A separate experiment was performed with the synthetic mixture. The analysis of synthetic mixture solution prepared above yielded percent recoveries which ranged of 94.78–122.1 with standard deviation of 0.98–1.46 in all the cases. The results of this study are presented in Table 4 indicating that the inactive ingredients did not interfere in the assay. These results further demonstrate the accuracy as well as the precision of the proposed methods.

#### 3.2.4. Application to Formulations

In order to evaluate the analytical applicability of the proposed methods to the quantification of FMT in commercial tablets, the results obtained by the proposed methods were compared to those of the reference method [2] by applying Student's *t*-test for accuracy and *F*-test for precision. The results (Table 5) show that the Student's *t*- and *F*-values at 95% confidence level are less than the theoretical values, which confirmed that there is a good agreement between the results obtained by the proposed methods and the reference method with respect to accuracy and precision.

#### 3.2.5. Recovery Studies

The accuracy and validity of the proposed methods were further ascertained by performing recovery studies. Preanalysed tablet powder was spiked with pure FMT at three concentration levels (50, 100, and 150% of that in tablet powder), and the total was found by the proposed methods. In all cases, the added FMT recovery percentage values ranged from 97.78 to 103.9% with standard deviation of 1.13–0.92 (Table 6) indicating that the recovery was good and that the coformulated substance did not interfere in the determination.

## 4. Conclusion

The methods described in this paper are simple, relatively specific, accurate, and precise for the determination of FMT. In particular, the proposed direct titrimetry is the simplest of all the methods reported so far for famotidine. The chromatographic techniques [3–8], although sensitive, require judicious control of pH of the mobile phase besides requiring expensive and sophisticated instruments. A large volume of solvents is required for these techniques, which are expensive, hazardous to health, and harmful to the environment. The methods based on such techniques as potentiometry [9], voltammetric [10], and polarography [13] require rigid pH control. The reliability and precision of the results by polarography [13] depend on the capillary characteristics which are often not reproducible. Even though the spectrofluorimetric methods [11, 12] are very sensitive, they use organic solvent which is not always desirable. The reported visible spectrophotometric methods based on ion-pair complexation reaction [17] require liquid-liquid extraction step, strict pH control, and large amounts of high purity solvents, which are often hazardous and result in the production of toxic lab waste. Other methods based on charge-transfer complexation reactions [18–21], complexation reaction using reagents like copper (II) chloride [22], cupric acetate [23], palladium (II) [24], condensation reaction [25], and redox reactions [15, 16, 26–30] suffer from disadvantages like poor sensitivity [15, 16, 18–28, 30], use of organic solvents [18–21, 25], strict pH control [16, 24], use of expensive reagent [19], boiling step [25, 29], and besides suffering from narrow linear range of applicability [22]. 

In contrast to the above-published methods, the present methods are sensitive, simple, and using ecofriendly chemicals, free from unwelcome steps such as heating or extraction and also from critical pH conditions. The present methods have wide linear dynamic ranges and can measure concentrations down to 0.8 and 0.3 *μ*g mL^−1^ with good precision and accuracy. The proposed methods are most sensitive compared to other reported visible spectrophotometric methods which are confirmed by the molar absorptivity values of 4.20 × 10^4^ and 1.09 × 10^5^ L mol^−1^ cm^−1^. The relative cheapness of apparatus and reagents demonstrate their advantageous characteristics. The methods are also useful due to high tolerance limit for common excipients found in drug formulations. These merits coupled with the use of simple and inexpensive instrument make the proposed methods acceptable in quality control laboratories for routine use.

## Figures and Tables

**Table 1:** Comparison of the performance characteristic of the existing spectrophotometric methods with the proposed methods.

Sl. No.	Reagent/s used	Methodology	Linear range (*μ*g mL^−1^) and molar absorptivity (l mol^−1^ cm^−1^)	Remarks	Reference
(1)	Chloramine-T and metol	Red-colored product was measured at 520 nm	0.0–40 (2.78 × 10^3^)	Less sensitive, metol solution is not stable, and even the blank absorbance is high	[15]
(2)	Potassium iodate (1) Dichlorofluorescein (2) Carbontetrachloride	The absorbance of the red-colored solutions was measured at 520 nm	(1) 2.5–25 (8.40 × 10^3^) (2) 200–1400 (1.16 × 10^2^)	Require strict pH control, and the sensitivity of the method which involves extraction was very less	[16]
(3)	(a) Bromocresol green (b) Bromothymol blue	Ion-pair complex measured at 420 nm	2.0–23 (*ϵ* = 5.0 × 10^3^) 0.7–8.1 (*ϵ* = 1.2 × 10^4^)	Sensitive but required close pH control and extraction use of organic solvent	[17]
(4)	(a) Chloranil (b) DDQ (c) DCNP	The increase in the absorbance is measured at 458, 460, and 425 nm, respectively	50–500	Poor sensitivity and use of organic solvent	[18]
(5)	Tetracyanoquinodimethane	Green radical anion measured at 840 nm	1.0–7.0	Uses expensive reagents and organic solvents	[19]
(6)	(a) *P*-chloranilic acid in methanol (b) *P*-chloranilic acid in acetonitrile	C-T complex measured at 525 nm	(a) 16000–110000 (b) 25–240	Less sensitive, uses acetonitrile which is very expensive	[20, 21]
(7)	Copper (II) chloride in methanolic medium	Blue-colored complex measured at 660 nm	200–1200 (*ϵ* = 1.11 × 10^2^)	Less sensitive, narrow linear range, and use of organic solvent	[22]
(8)	Cupric acetate	Complex measured at 630 nm	50–1250	Poor sensitivity	[23]
(9)	Palladium (II)	Yellow-colored complex measured at 345 nm in the p^H^ range 2.23–8.50	17–200	Sensitive but required close pH control	[24]
(10)	Ninhydrin in methanolic medium	Blue-colored product measured at 590 nm.	5.0–30.0	Requires heating in a boiling water bath and use of organic solvent	[25]
(11)	Sodium nitroprusside	Orange species formed in alkaline medium measured at 498 nm	50–500	Poor sensitivity	[26]
(12)	Brominating mixture	Yellow-colored developed measured at 350 nm	40–200	Less sensitive and measured at shorter wavelength	[27]
(13)	F-C reagent	Blue-colored product measured at 650 nm	16–48	Less selective	[28]
(14)	Fe (III) with (a) 1,10-phenanthroline (b) 2, 2-bipyridyl	(a) Ferroin measured at 510 nm (b) Fe (II)-bipyridyl complex measured at 520 nm	(a) 2–12 (*ϵ* = 2.9 × 10^4^) (b) 8–16 (*ϵ* = 1.6 × 10^4^)	Requires heating and longer reaction time	[29]
(15)	NBS with *p*-aminophenol	The decrease in the absorption intensity of the colored product measured at 552 nm	6.0–22.0	Two-step reaction, longer reaction time, and narrow linear range	[30]
(16)	NBS (a) indigo carmine (b) neutral red	Resulting colored products peaking at 610 nm and 530 nm	0.8–6.0 (*ϵ* = 4.199 × 10^4^) 0.3–3.0 (*ϵ* = 1.089 × 10^5^)	Highly sensitive, no heating or extraction step, inexpensive instrumental setup, use of ecofriendly chemicals, and aqueous system	Present methods

NBS: N-bromosuccinimide; FC: Folin-Ciocalteu; DDQ: dichloro dicyano benzoquinone; DCNP: dichloronitrophenol.

**Table 2:** Sensitivity and regression parameters.

Parameter	Method C	Method D
_max_,*λ* nm	610	530
Linear range, *μ*g mL^−1^	0.75–6.0	0.3–3.0
Molar absorptivity(*ε*), L mol^−1^ cm^−1^	4.199 × 10^4^	1.089 × 10^5^
Sandell sensitivity^a^, *μ*g cm^−2^	0.008	0.003
Limit of detection (LOD), *μ*g mL^−1^	0.05	0.02
Limit of quantification (LOQ), *μ*g mL^−1^	0.16	0.06
Regression equation, Y^b^		
Intercept (*a*)	0.011	0.02
Slope (*b*)	0.122	0.303
Standard deviation of a (*S*_*a*_)	0.048	0.063
Standard deviation of b (*S*_*b*_)	0.009	0.023
Regression coefficient (*r*)	0.996	0.999

^a^Limit of determination as the weight in *μ*g per mL of solution, which corresponds to an absorbance of A = 0.001 measured in a cuvette of cross-sectional area 1 cm^2^ and l = 1 cm. ^b^ *Y* = *a* + *bX*, where *Y* is the absorbance, *X* is concentration in *μ*g mL^−1^, *a* is intercept, and *b* is slope.

**Table 3:** Evaluation of intraday and interday accuracy and precision.

Method^a^	FMT taken	Intraday accuracy and precision			Interday accuracy and precision		
		FMT found	%RE	%RSD	FMT found	%RE	%RSD
Titrimetry method A	3.0	3.08	2.66	1.06	3.10	3.3	1.32
	6.0	6.1	1.67	1.01	6.13	2.17	0.97
	9.0	8.75	2.77	0.95	8.72	3.11	1.18
Method B	1.0	1.02	2.01	1.02	0.97	3.01	1.12
	5.0	4.94	1.22	0.98	4.89	2.22	1.34
	7.0	6.92	1.14	1.11	6.87	1.86	1.29
Spectrophotometry method C	3.0	3.07	2.33	0.92	3.10	3.33	1.15
	4.5	4.59	2.00	1.14	4.61	2.44	1.28
	6.0	5.8	3.33	1.02	5.82	3.01	1.11
Method D	0.6	0.62	3.33	1.25	0.58	3.33	1.35
	1.8	1.85	2.78	1.21	1.76	2.22	1.29
	3.0	2.94	2.02	1.13	3.10	3.33	1.17

RE: Relative error and RSD: Relative standard deviation.

^a^In titrimetry, FMTs taken/found are in mg and they are *μ*g mL^−1^  in spectrophotometry.

**Table 4:** Recovery of the drug from synthetic mixture.

Method	FMT in synthetic mixture taken^a^	FMT recovered^b^ (percent ± SD)
Method A	2.0	105.4 ± 1.13
	5.0	99.17 ± 1.21
	9.0	96.54 ± 1.08
Method B	2.0	96.01 ± 1.14
	4.0	95.93 ± 1.26
	6.0	94.78 ± 1.46
Method C	3.0	101.0 ± 1.04
	4.5	100.8 ± 1.01
	6.0	96.82 ± 0.98
Method D	0.6	117.3 ± 1.19
	1.8	122.1 ± 1.05
	3.0	113.7 ± 1.15

^a^mg in titrimetry and *μ*g mL^−1^  in spectrophotometry.

^b^Mean value of five determinations.

**Table 5:** Results of analysis of tablets by the proposed methods.

Tablet brand name	Label claim, mg/tablet	Found^a^ (percent of label claim ± SD)				
		Reference method	Titrimetry		Spectrophotometry	
			Method A	Method B	Method C	Method D
Topcid 20^b^	20	102.5 ± 0.93	101.21 ± 1.19 *t* = 1.92 *F *= 1.63	101.24 ± 0.87 *t *= 2.21 *F *= 1.14	103.5 ± 0.91 *t *= 1.72 *F *= 1.04	103.7 ± 1.07 *t *=1.90 *F *= 1.32
Famocid 20^c^	20	101.3 ± 1.02	99.56 ± 1.17 *t *= 2.51 *F *= 0.76	99.86 ± 1.06 *t *= 2.19 *F *= 0.93	102.7 ± 1.09 *t *=2.09 *F *=1.11	102.5 ± 0.95 *t *= 1.92 *F *= 0.98

^a^Mean value of five determinations. ^b^Torrent Pharmaceuticals Ltd., H. P, India; ^c^Sun Pharmaceuticals Industries, Jammu, India.

The value of *t* (tabulated) at 95% confidence level and for four degrees of freedom is 2.77.

The value of *F* (tabulated) at 95% confidence level and for four degrees of freedom is 6.39.

**Table 6:** Accuracy assessment by recovery experiments.

Method	Tablet studied	FMT in tablet^a^	Pure FMT added^a^	Total found^a^	Pure FMT recovered^b^ percent ± SD
Titrimetry method A	Topcid 20	3.0	1.5	4.52	101.3
		3.0	3.0	5.97	99.0
		3.0	4.5	7.40	97.78
Method B	Topcid 20	2.0	1.0	2.99	99.0
		2.0	2.0	4.05	102.5
		2.0	3.0	4.97	98.89
Spectrophotometry method C	Topcid 20	1.5	0.75	2.27	102.7
		1.5	1.5	3.04	103.1
		1.5	2.25	3.81	102.7
Method D	Topcid 20	1.2	0.6	1.82	103.3
		1.2	1.2	2.43	102.5
		1.2	1.8	3.07	103.9

^a^mg in titrimetry and *μ*g mL^−1^  in spectrophotometry.

^b^Mean value of three measurements.
